# Supplementary material for: De novo activating mutations drive clonal evolution and enhance clonal fitness in KMT2A-rearranged leukemia
Source: Nat Commun. 2018 May 2;9:1770. doi: 10.1038/s41467-018-04180-1 (PMC5932012; doi:10.1038/s41467-018-04180-1)
Supplement: Supplementary file 1 — Supplementary Information [file 41467_2018_4180_MOESM1_ESM.pdf]

Supplementary Information

De novo activating mutations drive clonal evolution and enhance clonal fitness in *KMT2A*-rearranged leukemia

Hyrenius-Wittsten et al.

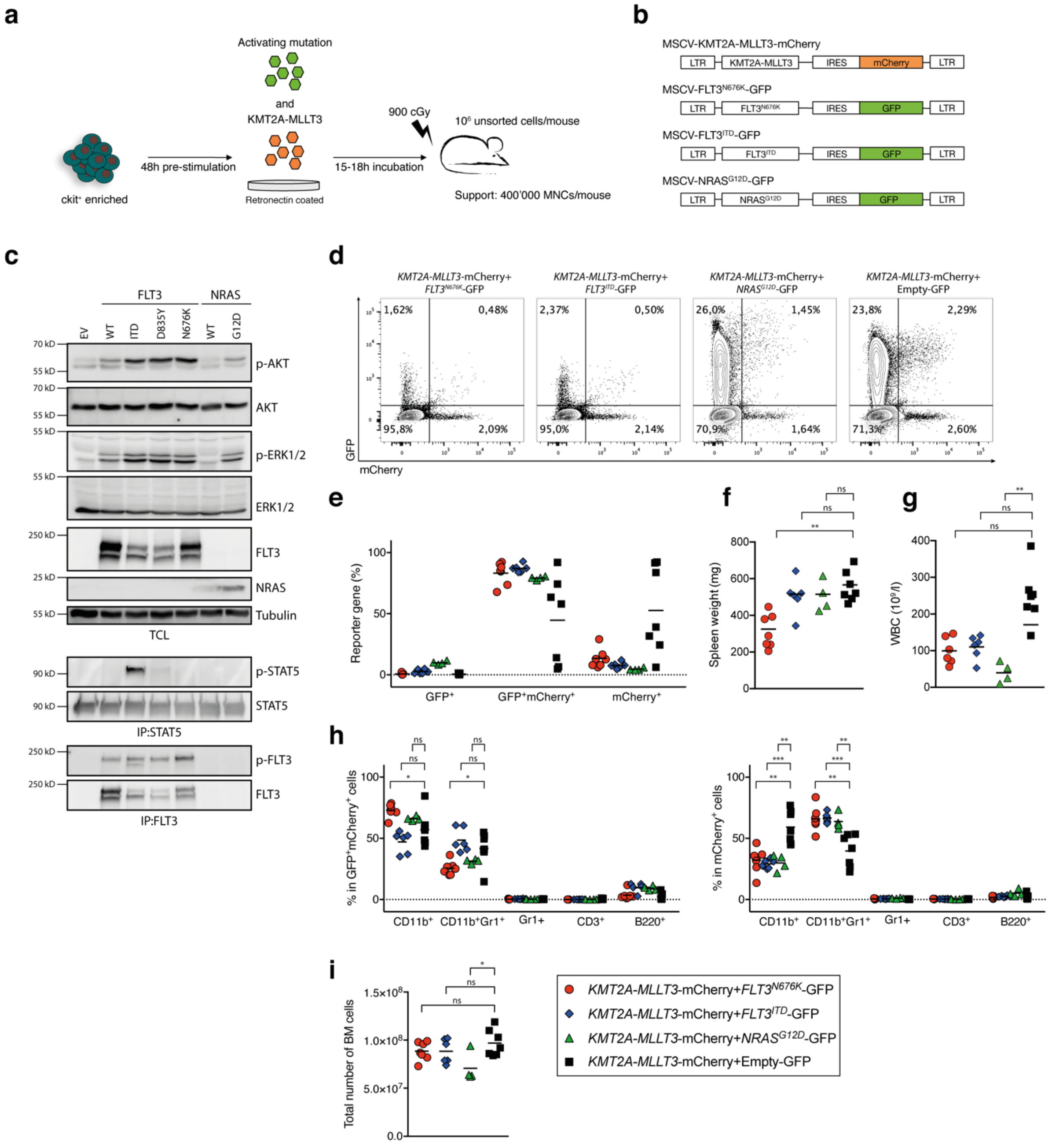

Supplementary Figure 1. Activating mutations in FLT3- and RAS-signaling gain clonal dominance at AML onset. (**a**) Schematic experimental outline of the retroviral bone marrow transplantation assay. (**b**) The retroviral constructs MSCV-KMT2A-MLLT3-IRES-mCherry, MSCV-FLT3^{N676K}-IRES-GFP, MSCV-FLT3^{ITD}-IRES-GFP and MSCV-NRAS^{G12D}-IRES-GFP. (**c**) Serum-starved Ba/F3 cells transduced with Empty-GFP, *FLT3^{WT}*-GFP, *FLT3^{ITD}*-GFP, *FLT3^{D835Y}*-GFP, *FLT3^{N676K}*-GFP, *NRAS^{WT}*-GFP, or *NRAS^{G12D}*-GFP were assessed for phosphorylation of AKT, ERK1/2, FLT3, and STAT5. (**d**) Co-transduction efficiency as assessed by flow cytometry 48 hours post-transduction. (**e**) Distribution of GFP and mCherry expressing cells in bone marrow (BM) of moribund primary recipient mice. (**f**) Spleen weight

and (**g**) white blood cell count (WBC) for primary recipient mice at the time of sacrifice. (**h**) Mature lineage distribution in $GFP^{+}mCherry^{+}$ and $mCherry^{+}$ BM cells using the myeloid markers CD11b and Gr1 and lymphoid markers B220 and CD3, showed a majority of myeloid cells for all groups. (**i**) Total number of BM cells (collected from 2 illiacs, 2 tibias, and 1 femur) at sacrifice. *$P \leq 0.05$, **$P \leq 0.01$, ***$P \leq 0.001$, ns=not significant

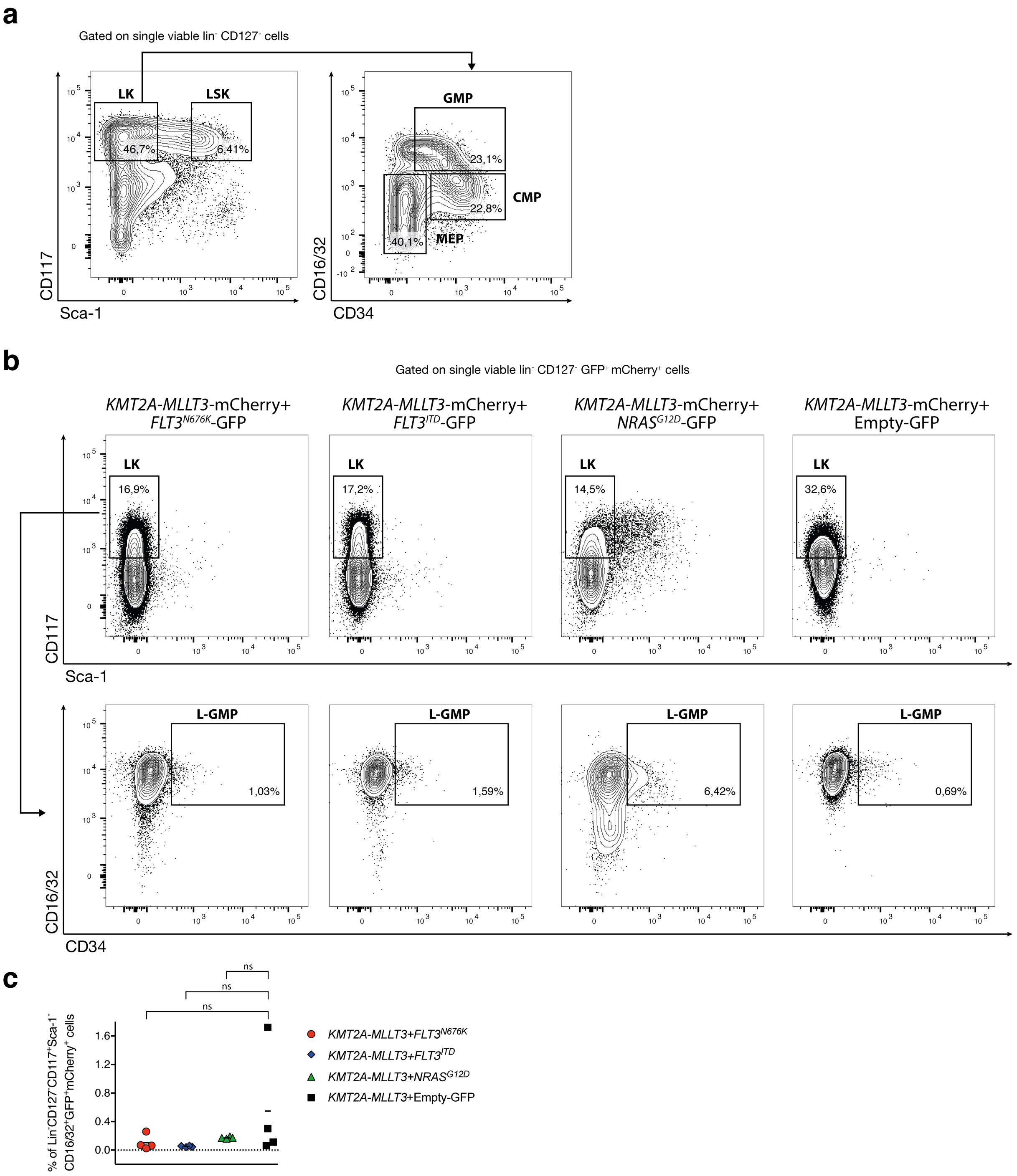

Supplementary Figure 2. Activating mutations do not influence immature leukemic populations. (**a**) Gating strategy for progenitor compartments Lin$^-$CD127$^-$CD117$^+$Sca-1$^-$ (LK), Lin$^-$CD127$^-$CD117$^+$Sca-1$^+$ (LSK), Lin$^-$CD127$^-$CD117$^+$Sca-1$^-$CD16/32$^{lo/-}$CD34$^-$ (MEP), Lin$^-$CD127$^-$CD117$^+$Sca-1$^-$CD16/32^{lo}CD34$^+$ (CMP) and Lin$^-$CD127$^-$CD117$^+$Sca-1$^-$CD16/32$^+$CD34$^+$ (GMP) in CD117 enriched wild-type BM. Left plot is gated on single viable Lin$^-$/CD127$^-$ cells. (**b**) Representative flow cytometry contour plots of leukemic BM of moribund mice, revealing the presence of Lin$^-$CD127$^-$CD117$^+$Sca-1$^-$CD16/32$^+$CD34$^+$ leukemic GMP-like cells (L-GMP). Top plots are gated on single viable Lin$^-$CD127$^-$GFP$^+$mCherry$^+$ cells.

(**c**) Distribution of L-GMP within $GFP^{+}mCherry^{+}$ cells of moribund mice (n = 4 for all groups). ns=not significant

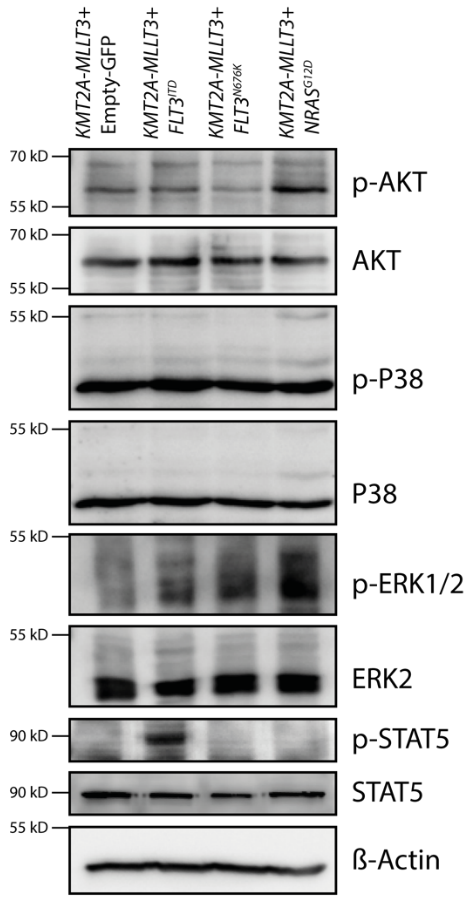

Supplementary Figure 3. *KMT2*-R leukemias with activating mutations show activation of distinct signaling pathways. Serum-starved *ex vivo* cultured primary leukemic cells were assessed for phosphorylation of AKT, P38, ERK1/2, and STAT5.

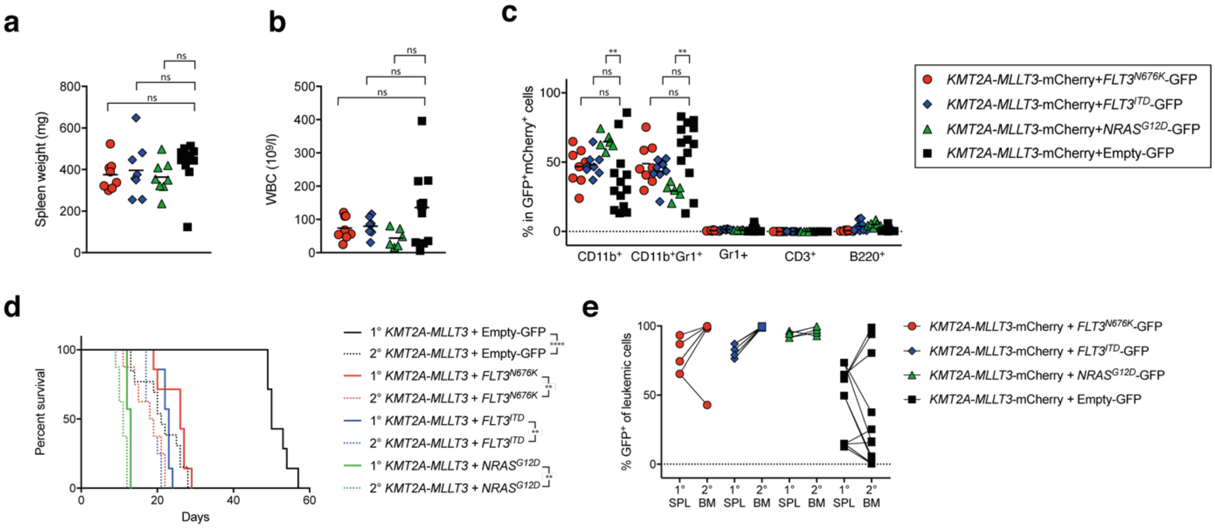

Supplementary Figure 4. Activating mutations are further enriched in secondary *KMT2A-MLLT3* recipients. (**a**) Spleen weight and (**b**) WBC for secondary recipient mice at sacrifice. (**c**) Mature lineage distribution in $GFP^{+}mCherry^{+}$ BM cell of moribund secondary recipient mice. (**d**) Survival of primary recipients (1°; intact line) showed a significantly reduced disease latency for all groups as compared to their respective secondary recipients (2°; dotted line) (*P*=0.006 for *KMT2A-MLLT3+FLT3*^{N676K}, *P*=0.0011 for *KMT2A-MLLT3+FLT3*^{ITD}, *P*=0.0034 for *KMT2A-MLLT3+NRAS*^{G12D}, and *P*<0.0001 for *KMT2A-MLLT3*+Empty-GFP. Mantel-Cox log-rank test). (**e**) Progression of GFP^{+} cells within the $mCherry^{+}$ leukemic population between primary (1°) spleen (SPL) and secondary (2°) BM showed a continued enrichment of GFP^{+} cells for *KMT2A-MLLT3*-mCherry+*FLT3*^{N676K}-GFP cells (*P*=0.0770. Paired t-test), *KMT2A-MLLT3*-mCherry+*FLT3*^{ITD}-GFP cells (paired t-test; *P*<0.0001), *KMT2A-MLLT3*-mCherry+*NRAS*^{G12D}-GFP cells (*P*=0.0598. Paired t-test) but not for *KMT2A-MLLT3*-mCherry+Empty-GFP cells (*P*=0.2209. Paired t-test). ***P*≤0.01, *****P*<0.0001, ns=not significant

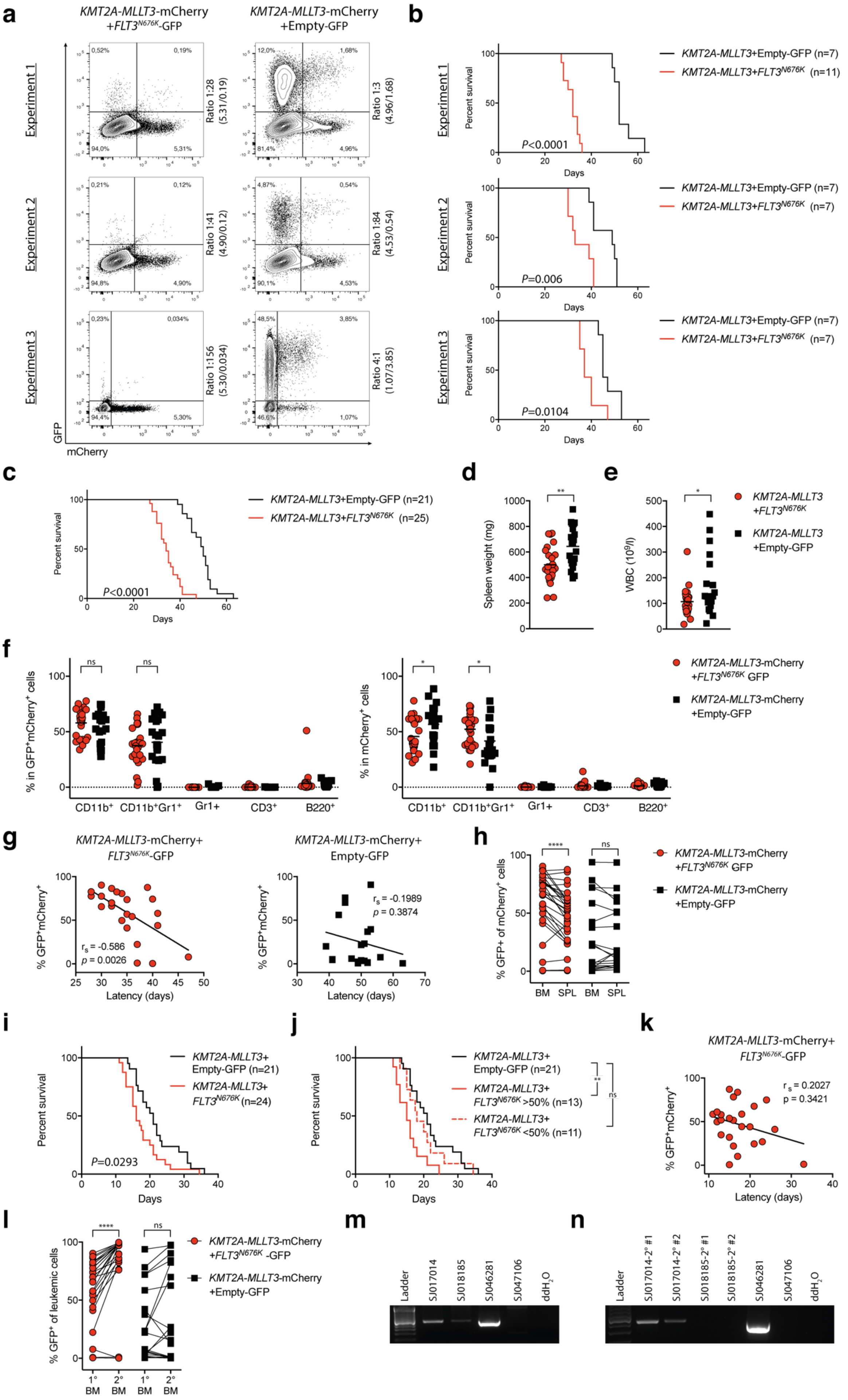
a
KMT2A-MLLT3-mCherry +FLT3N676K-GFP
KMT2A-MLLT3-mCherry +Empty-GFP
Experiment 1
Experiment 2
Experiment 3
Ratio 1:28 (5.31/0.19)
Ratio 1:3 (4.96/1.68)
Ratio 1:41 (4.90/0.12)
Ratio 1:84 (4.53/0.54)
Ratio 1:156 (5.30/0.034)
Ratio 4:1 (1.07/3.85)
GFP
mCherry
b
KMT2A-MLLT3+Empty-GFP (n=7)
KMT2A-MLLT3+FLT3N676K (n=11)
KMT2A-MLLT3+FLT3N676K (n=7)
Percent survival
Days
P<0.0001
P=0.006
P=0.0104
c
KMT2A-MLLT3+Empty-GFP (n=21)
KMT2A-MLLT3+FLT3N676K (n=25)
d
Spleen weight (mg)
e
WBC (10⁹/l)
KMT2A-MLLT3 +FLT3N676K
KMT2A-MLLT3 +Empty-GFP
f
% in GFP+mCherry+ cells
% in mCherry+ cells
CD11b+ CD11b+Gr1+ Gr1+ CD3+ B220+
KMT2A-MLLT3-mCherry +FLT3N676K GFP
KMT2A-MLLT3-mCherry +Empty-GFP
g
KMT2A-MLLT3-mCherry+ FLT3N676K-GFP
KMT2A-MLLT3-mCherry+ Empty-GFP
% GFP+mCherry+
rs = -0.586
p = 0.0026
rs = -0.1989
p = 0.3874
Latency (days)
h
% GFP+ of mCherry+ cells
BM SPL BM SPL
i
KMT2A-MLLT3+ Empty-GFP (n=21)
KMT2A-MLLT3+ FLT3N676K (n=24)
P=0.0293
j
KMT2A-MLLT3+ Empty-GFP (n=21)
KMT2A-MLLT3+ FLT3N676K >50% (n=13)
KMT2A-MLLT3+ FLT3N676K <50% (n=11)
k
KMT2A-MLLT3-mCherry+ FLT3N676K-GFP
rs = 0.2027
p = 0.3421
l
% GFP+ of leukemic cells
1° BM 2° BM 1° BM 2° BM
KMT2A-MLLT3-mCherry +FLT3N676K -GFP
KMT2A-MLLT3-mCherry +Empty-GFP
m
Ladder SJ017014 SJ018185 SJ046281 SJ047106 ddH2O
n
Ladder SJ017014-2° #1 SJ017014-2° #2 SJ018185-2° #1 SJ018185-2° #2 SJ046281 SJ047106 ddH2O

Supplementary Figure 5. Subclonal *FLT3*^{N676K} accelerates AML onset. (**a**) Co-transduction efficiency as assessed by flow cytometry 48 hours post-transduction. (**b,c**) Kaplan-Meier survival curves for three independent experiments with mice transplanted with either *KMT2A-MLLT3*-mCherry+*FLT3*^{N676K}-GFP or *KMT2A-MLLT3*-mCherry+Empty-GFP (**b**) separated for each experiment and (**c**) combined. (**d**) Spleen weight and (**e**) WBC for diseased primary recipient mice. (**f**) Mature lineage distribution in GFP^{+}mCherry^{+} and mCherry^{+} BM cells using the myeloid markers CD11b and Gr1, and lymphoid markers B220 and CD3. (**g**) Spearman correlation between GFP^{+}mCherry^{+} (%) in BM at time of sacrifice and disease latency showing a significant correlation for *KMT2A-MLLT3+FLT3*^{N676K} (r_s=-0.586, *P*=0.0026. Spearman´s rank correlation coefficient) but not for *KMT2A-MLLT3*+Empty-GFP (r_s=-0.1989, *P*=0.3874. Spearman´s rank correlation coefficient). (**h**) Frequency of GFP^{+} in mCherry^{+} cells in the BM and spleen (SPL) of primary recipients, showing a significantly lower frequency of GFP^{+} in mCherry^{+} cells in the SPL for *KMT2A-MLLT3*-mCherry+*FLT3*^{N676K}-GFP (*P*<0.0001. Paired t-test) but not for *KMT2A-MLLT3*-mCherry+Empty-GFP (*P*=0.2485. Paired t-test). (**i**) Kaplan-Meier curves for secondary recipients transplanted with primary leukemic splenocytes showed a small difference in disease latency between *KMT2A-MLLT3+FLT3*^{N676K} and *KMT2A-MLLT3*+Empty-GFP. (**j**) Kaplan-Meier curves for secondary recipients with *KMT2A-MLLT3+FLT3*^{N676K} divided based on presence of dominant- (>50%) and subclonal (<50%) *FLT3*^{N676K} containing cells in primary leukemic splenocytes (dominant clone *P*=0.0018 and subclone *P*=0.3891. Mantel-Cox log-rank test). (**k**) Evolution of *FLT3*^{N676K}-GFP^{+} cells within the mCherry^{+} leukemic population between primary (1°) BM and secondary (2°) BM showed a significant expansion of *KMT2A-MLLT3*-mCherry+*FLT3*^{N676K}-GFP cells (paired t-test; *P*<0.0001) but not of *KMT2A-MLLT3*-mCherry+Empty-GFP cells (*P*=0.1675. Paired t-test). (**l**) Spearman correlation between GFP^{+}mCherry^{+} (%) in BM at time of sacrifice and disease latency showed no significant correlation for *KMT2A-MLLT3+FLT3*^{N676K} (r_s=-0.2027, *P*=0.3421. Spearman´s rank correlation coefficient) in secondary recipients. (**m,n**) DNA PCR validation of small subclones (<1% of BM cells) *KMT2A-MLLT3+FLT3*^{N676K} for (**m**) SJ017014 (1.1% *FLT3*^{N676K} of leukemic cells) and SJ018185 (0.5% *FLT3*^{N676K} of leukemic cells) and (**n**) in their corresponding secondary recipients (2˚); positive control SJ046281 (83% *KMT2A-MLLT3+FLT3*^{N676K} of BM cells), negative control SJ047106 (*KMT2A-MLLT3*+Empty-GFP), and water control (ddH_2O). **P*≤0.05, ***P*≤0.01, *****P*≤0.0001, ns=not significant

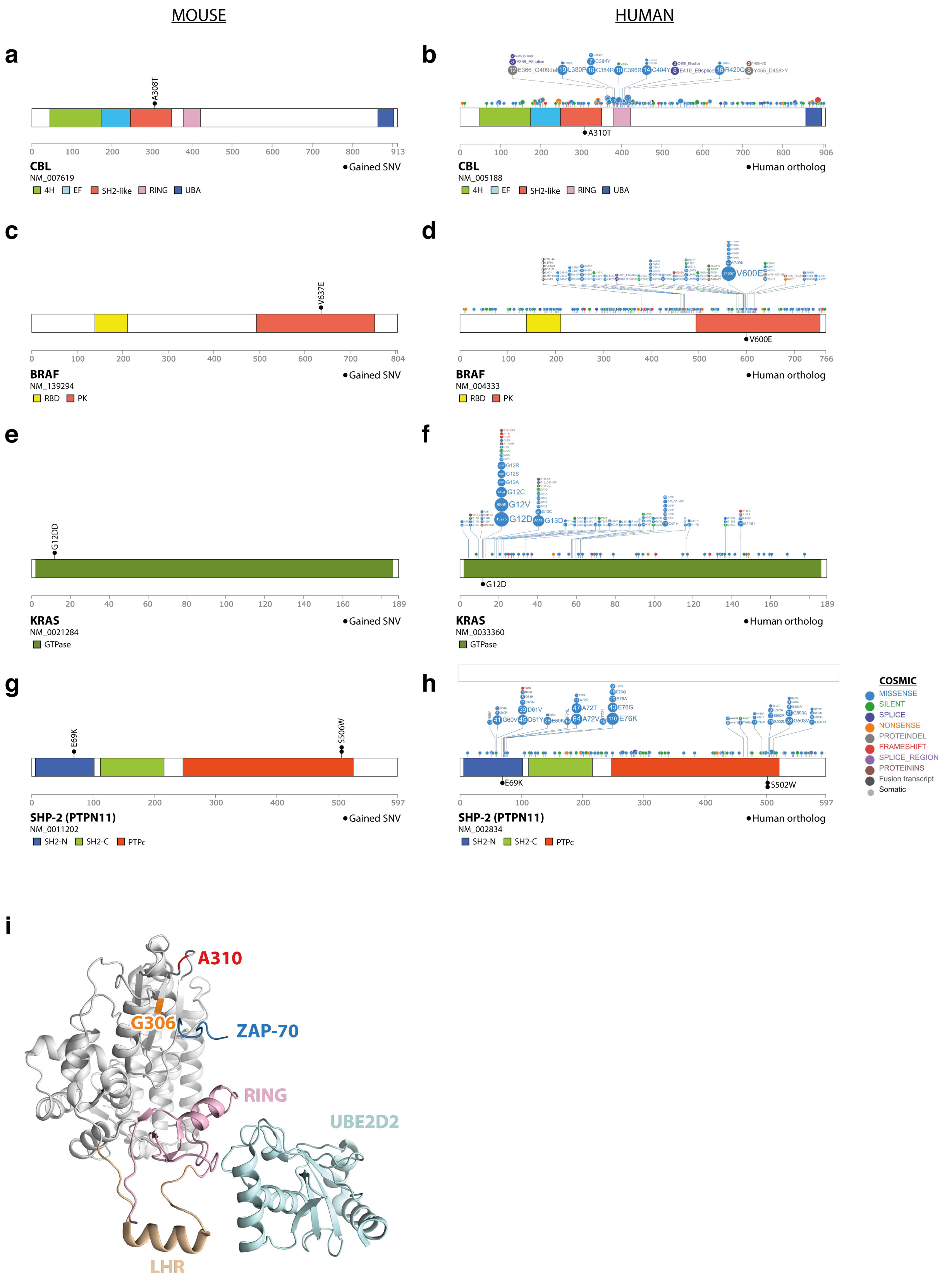

Supplementary Figure 6. ***KMT2A-MLLT3*** **cells acquire** ***de novo*** **mutations linked to active signaling.** Corresponding mouse protein positions for identified and validated *de novo* mutations (highlighted with black dot and line) and their human orthologous position

(highlighted with black dot and line) for (**a,b**) CBL, (**c,d**) BRAF, (**e,f**) KRAS, and (**g,h**) SHP-2 (PTPN11). Human protein orthologs are highlighted with reported human mutations in COSMIC[30] (http://cancer.sanger.ac.uk/cosmic). Proteins and COSMIC mutational charts were created using protein paint (pecan.stjude.org). (**i**) Ribbon representation of the human N-terminal SH2-containing tyrosine kinase-binding (TKB) domain of CBL, in an active state, bound to ZAP-70 peptide (blue) and the Ubiquitin-conjugating enzyme E2 D2 (UBE2D2; cyan) (PDBID:4A4B). Also highlighting the RING domain (pink), LHR (beige), and residue G306 (orange) and A310 (red).

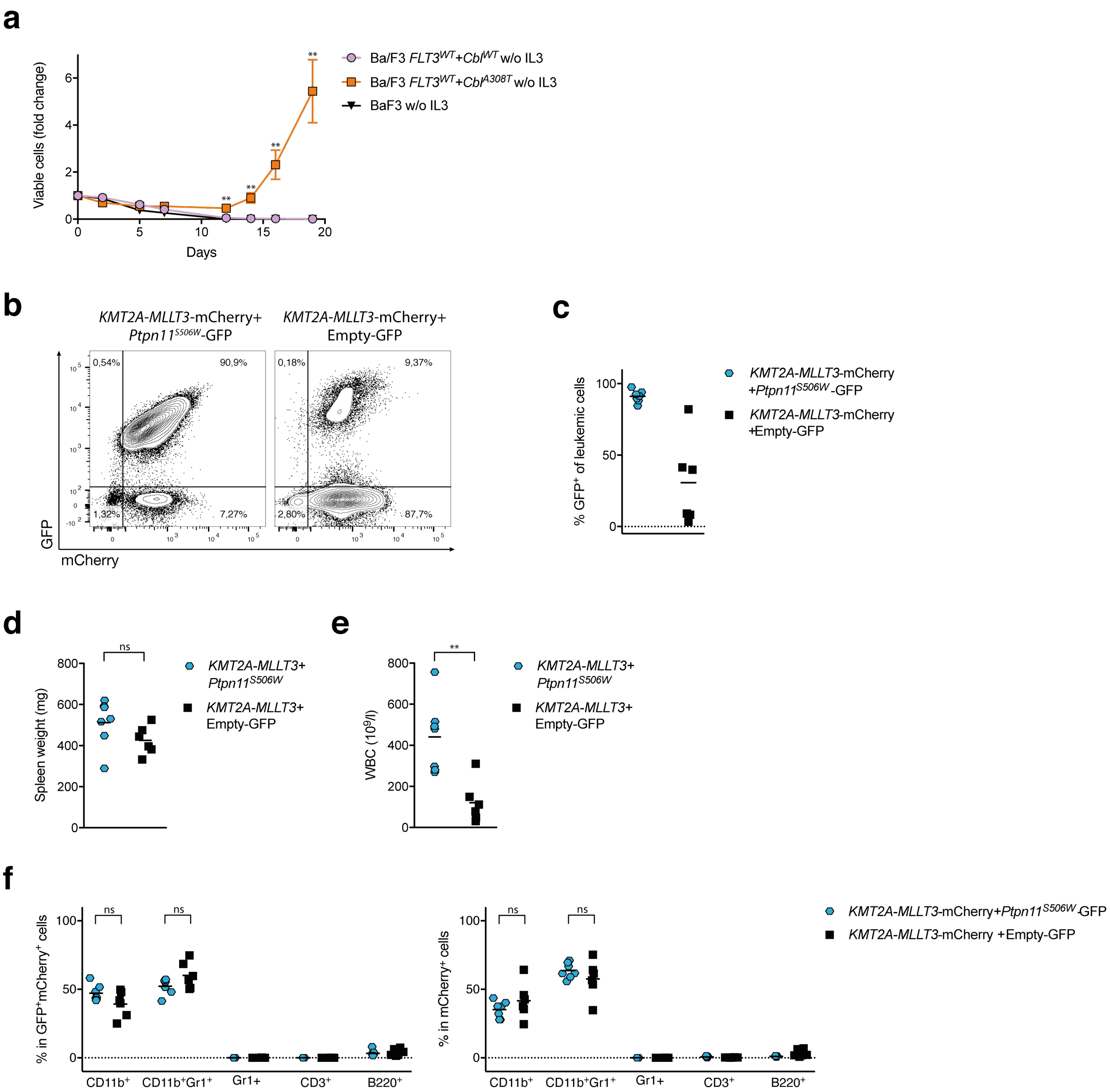

Supplementary Figure 7. Functional validation of *Cbl*^{A308T} and *Ptpn11*^{S506W}. (**a**) Ba/F3 cells co-transduced with *FLT3*^{WT} and *Cbl*^{A308T} showed significantly higher IL3-independent growth as compared to *FLT3*^{WT} and *Cbl*^{A308T}. Data shown as fold change of Ba/F3 cells expressing solely *FLT3*^{WT}. Error bars are s.d. (**b**) Representative flow cytometric contour plots of BM from sacrificed mice showing transgene distribution as determined by GFP and mCherry expression. (**c**) Distribution of GFP within mCherry expressing cells in BM of moribund primary recipient mice. (**d**) Spleen weight and (**e**) WBC for diseased primary recipient mice transplanted with either *KMT2A-MLLT3*+*Ptpn11*^{S506W} or *KMT2A-MLLT3*+Empty-GFP. (**f**) Mature lineage distribution in GFP^{+}mCherry^{+} and mCherry^{+} BM cells using the myeloid markers CD11b and Gr1 and lymphoid markers B220 and CD3. ***P*≤0.01, ns=not significant

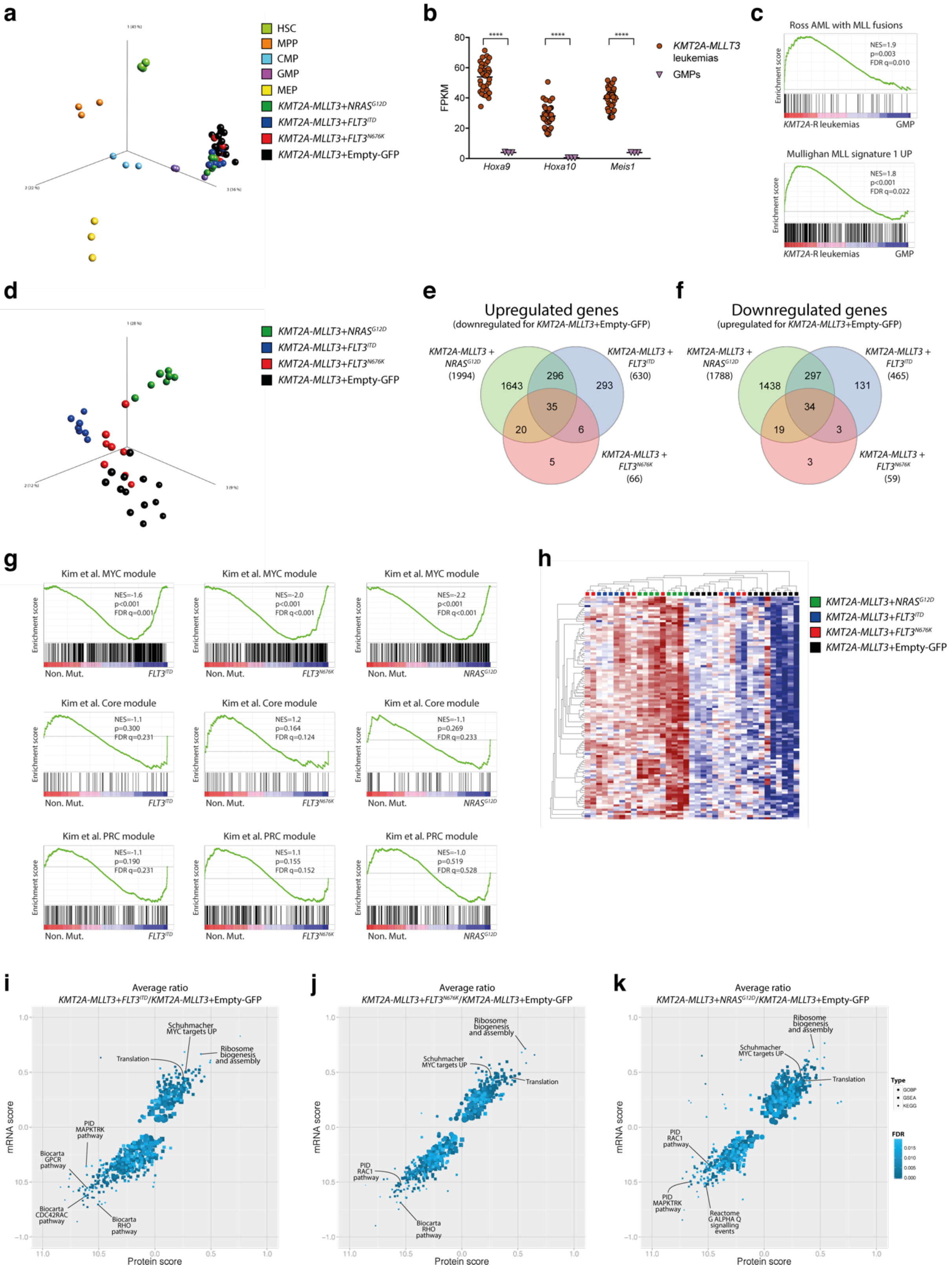

Supplementary Figure 8. Activating mutations enforce a Myc-centered transcriptional program. (**a**) Unsupervised (12705 variables) principal component analysis based on sorted normal mouse hematopoietic progenitors[1] of hematopoietic stem cells (HSC), multipotent progenitors (MPP), megakaryocyte erythroid progenitor (MEP), common myeloid progenitor

(CMP), and granulocyte macrophage progenitors (GMP) (**Supplementary Table 4**). Leukemias with *KMT2A-MLLT3+NRAS*^{G12D}, *KMT2A-MLLT3+FLT3*^{ITD} and *KMT2A-MLLT3+FLT3*^{N676K} were inserted into the same PCA (still based solely on the normal populations), revealing that they are different from the normal populations but, given the principal components from the normal cells, sharing most resemblance to GMPs. (**b**) Expression (FPKM) of *Hoxa9, Hoxa10,* and *Meis1* in primary *KMT2A-MLLT3* with and without *NRAS*^{G12D}*, FLT3*^{ITD}*,* or *FLT3*^{N676K} and normal GMPs. (**c**) GSEA revealed that e.g., pediatric AML *KMT2A*-signatures are enriched in the mouse leukemias as compared to the normal GMP population. (**d**) Unsupervised (5000 variables) principal component analysis of primary mouse leukemias with *KMT2A-MLLT3+NRAS*^{G12D}*, KMT2A-MLLT3+FLT3*^{ITD}, *KMT2A-MLLT3+FLT3*^{N676K}, and *KMT2A-MLLT3*+Empty-GFP. (**e,f**) Venn diagrams showing (**e**) upregulated- and (**f**) downregulated genes for *KMT2A-MLLT3+FLT3*^{ITD}, *KMT2A-MLLT3+FLT3*^{N676K}, and *KMT2A-MLLT3+NRAS*^{G12D} as compared to *KMT2A-MLLT3*+Empty-GFP (FDR≤0.01); note that upregulated genes in (**e**) are downregulated for *KMT2A-MLLT3*+Empty-GFP and downregulated genes in (**f**) are upregulated for *KMT2A-MLLT3*+Empty-GFP. (**g**) GSEA revealed enrichment of the MYC module, but not Core- or polycomb (PRC) modules[2] for mouse *KMT2A-MLLT3* leukemias with *FLT3*^{ITD}, *FLT3*^{N676K}, or *NRAS*^{G12D}. (**h**) Unsupervised hierarchical clustering (104 variables) of mouse *KMT2A*-R leukemias with or without *FLT3*^{ITD}, *FLT3*^{N676K}, or *NRAS*^{G12D} based on shared MYC module leading edge genes (**Supplementary Figure 8g**). (**i-k**) 2D enrichment analysis of the average ratio from transcriptomic and proteomic data between (**i**) *KMT2A-MLLT3+FLT3*^{ITD} and *KMT2A-MLLT3*+Empty-GFP, (**j**) *KMT2A-MLLT3+FLT3*^{N676K} and *KMT2A-MLLT3*+Empty-GFP, (**k**) *KMT2A-MLLT3+NRAS*^{G12D} and *KMT2A-MLLT3*+Empty-GFP. ****$P \leq 0.0001$

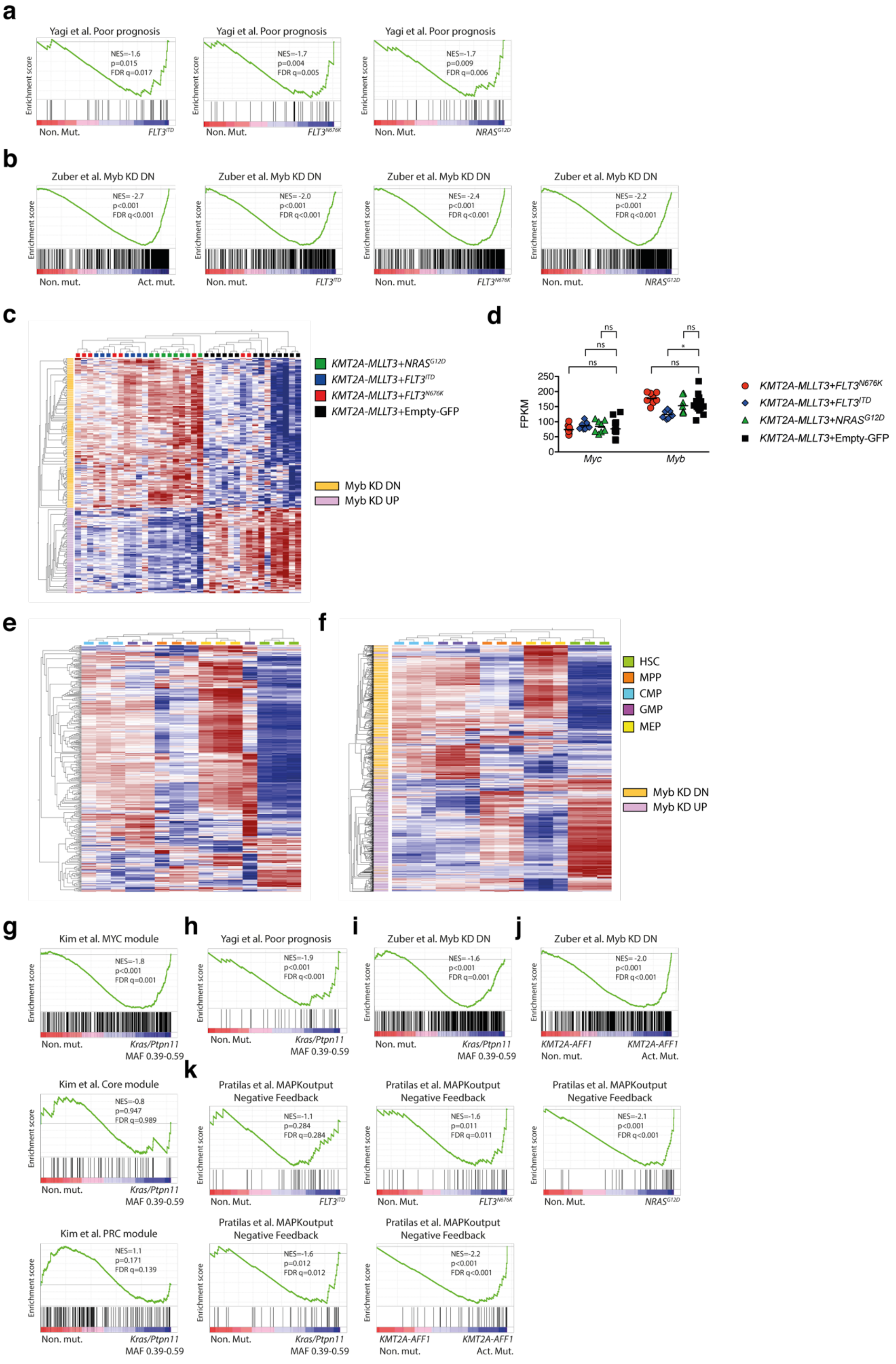
a
Yagi et al. Poor prognosis
NES=-1.6
p=0.015
FDR q=0.017
Enrichment score
Non. Mut.
FLT3ITD
Yagi et al. Poor prognosis
NES=-1.7
p=0.004
FDR q=0.005
Non. Mut.
FLT3N676K
Yagi et al. Poor prognosis
NES=-1.7
p=0.009
FDR q=0.006
Non. Mut.
NRASG12D
b
Zuber et al. Myb KD DN
NES= -2.7
p<0.001
FDR q<0.001
Non. mut.
Act. mut.
Zuber et al. Myb KD DN
NES= -2.0
p<0.001
FDR q<0.001
Non. mut.
FLT3ITD
Zuber et al. Myb KD DN
NES= -2.4
p<0.001
FDR q<0.001
Non. mut.
FLT3N676K
Zuber et al. Myb KD DN
NES= -2.2
p<0.001
FDR q<0.001
Non. mut.
NRASG12D
c
KMT2A-MLLT3+NRASG12D
KMT2A-MLLT3+FLT3ITD
KMT2A-MLLT3+FLT3N676K
KMT2A-MLLT3+Empty-GFP
Myb KD DN
Myb KD UP
d
FPKM
250
200
150
100
50
0
ns
ns
ns
ns
*
ns
Myc
Myb
KMT2A-MLLT3+FLT3N676K
KMT2A-MLLT3+FLT3ITD
KMT2A-MLLT3+NRASG12D
KMT2A-MLLT3+Empty-GFP
e
f
HSC
MPP
CMP
GMP
MEP
Myb KD DN
Myb KD UP
g
Kim et al. MYC module
NES=-1.8
p<0.001
FDR q=0.001
Non. mut.
Kras/Ptpn11
MAF 0.39-0.59
h
Yagi et al. Poor prognosis
NES=-1.9
p<0.001
FDR q<0.001
Non. Mut.
Kras/Ptpn11
MAF 0.39-0.59
i
Zuber et al. Myb KD DN
NES= -1.6
p<0.001
FDR q=0.001
Non. mut.
Kras/Ptpn11
MAF 0.39-0.59
j
Zuber et al. Myb KD DN
NES= -2.0
p<0.001
FDR q<0.001
KMT2A-AFF1
Non. mut.
KMT2A-AFF1
Act. Mut.
Kim et al. Core module
NES=-0.8
p=0.947
FDR q=0.989
Non. mut.
Kras/Ptpn11
MAF 0.39-0.59
k
Pratilas et al. MAPKoutput
Negative Feedback
NES=-1.1
p=0.284
FDR q=0.284
Non. Mut.
FLT3ITD
Pratilas et al. MAPKoutput
Negative Feedback
NES=-1.6
p=0.011
FDR q=0.011
Non. Mut.
FLT3N676K
Pratilas et al. MAPKoutput
Negative Feedback
NES=-2.1
p<0.001
FDR q<0.001
Non. Mut.
NRASG12D
Kim et al. PRC module
NES=1.1
p=0.171
FDR q=0.139
Non. mut.
Kras/Ptpn11
MAF 0.39-0.59
Pratilas et al. MAPKoutput
Negative Feedback
NES=-1.6
p=0.012
FDR q=0.012
Non. Mut.
Kras/Ptpn11
MAF 0.39-0.59
Pratilas et al. MAPKoutput
Negative Feedback
NES=-2.2
p<0.001
FDR q<0.001
KMT2A-AFF1
Non. mut.
KMT2A-AFF1
Act. Mut.

Supplementary Figure 9. Activating mutations enforce a *Myb*-centered transcriptional program. (**a**) GSEA revealed an enrichment of a pediatric AML poor prognosis signature[3] for mouse *KMT2A-MLLT3* leukemias with $FLT3^{ITD}$, $FLT3^{N676K}$, and $NRAS^{G12D}$. (**b**) GSEA revealed enrichment of a described Myb signature[4] in the presence of $FLT3^{ITD}$, $FLT3^{N676K}$, and/or $NRAS^{G12D}$ (Act. Mut.). (**c**) Unsupervised hierarchical clustering (215 variables) of mouse *KMT2A*-R leukemias with or without $FLT3^{ITD}$, $FLT3^{N676K}$, and/or $NRAS^{G12D}$ based on shared *Myb* Knock Down (KD) DN and *Myb* KD UP[4] leading edge genes (**Supplementary Figure 9b** and **Supplementary Data 17-19**). (**d**) Expression (FPKM) of *Myc* and *Myb* in mouse *KMT2A-MLLT3* leukemias with or without $FLT3^{ITD}$, $FLT3^{N676K}$, or $NRAS^{G12D}$. (**e,f**) Unsupervised hierarchical clustering of HSCs, MPPs, CMPs, GMPs, and MEPs based on the (**e**) MYC module[2]- (386 variables) and (**f**) *Myb* KD DN and *Myb* KD UP[4] (963 variables) genes (**Supplementary Data 15**). (**g-i**) GSEA revealed an enrichment of (**g**) the MYC module, but not Core- or polycomb (PRC) modules[2], (**h**) pediatric AML poor prognosis signature[3], and (**i**) a described *Myb* signature[4] for mouse *KMT2A-MLLT3* leukemias with *de novo* mutations in *Kras* and *Ptpn11* (MAF 0.39-0.59). (**j**) GSEA revealed enrichment of the *Myb* signature[4] in infant *KMT2A-AFF1* ALL patients with activating mutations (dominant or subclonal) as compared to those lacking such mutations. (**k**) GSEA revealed enrichment of a MEK/ERK output and negative feedback signature[5] for mouse *KMT2A-MLLT3* leukemias with $FLT3^{N676K}$, $NRAS^{G12D}$, and *Kras/Ptpn11* mutations (MAF 0.39-0.59) as well as for infant *KMT2A-AFF1* ALL patients with activating mutations (dominant or subclonal), but not for mouse *KMT2A-MLLT3* leukemias with $FLT3^{ITD}$. $*P \leq 0.05$, ns = not significant

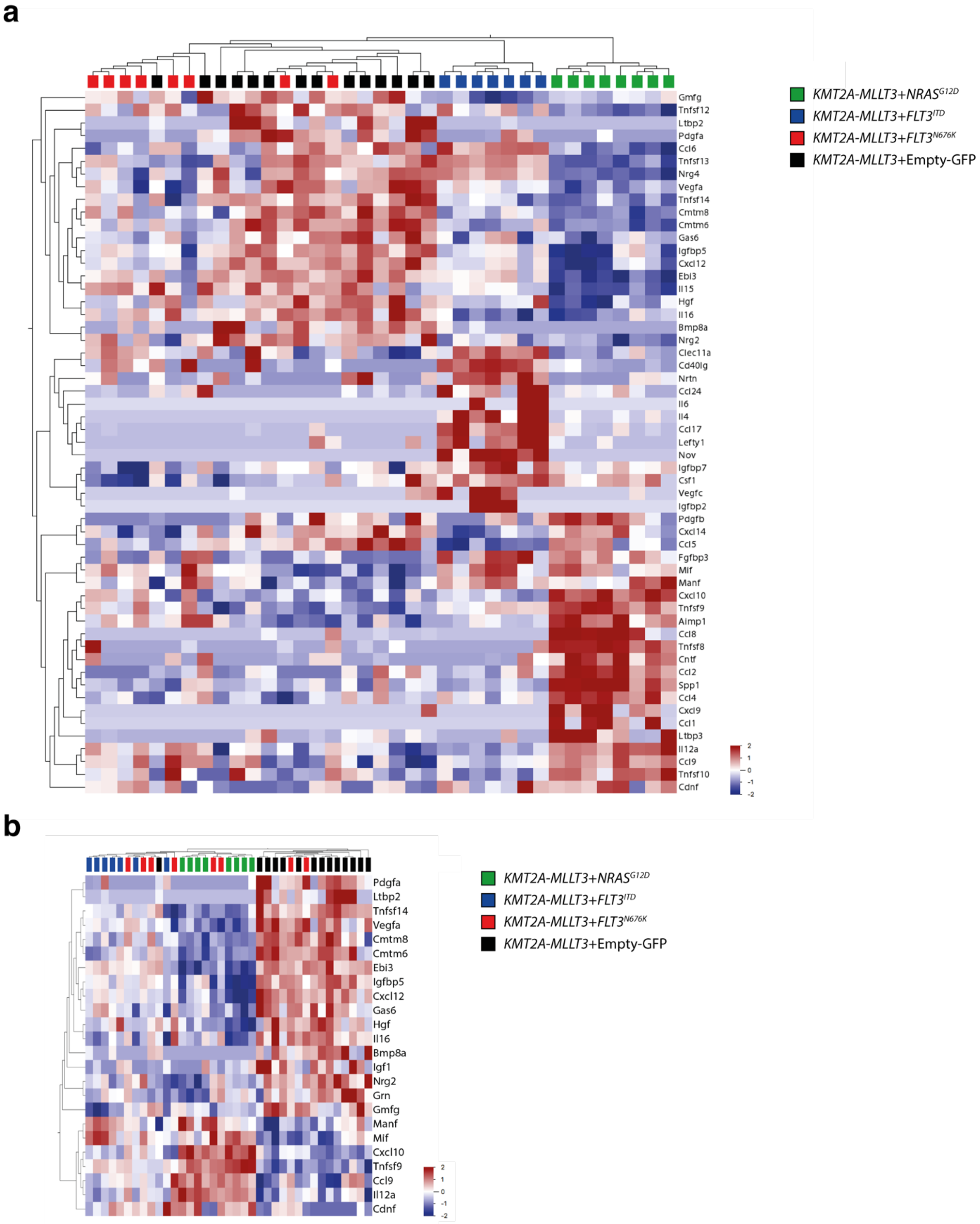

Supplementary Figure 10. Activating mutations influence cytokine- and growth factor expression. Hierarchical clustering after (**a**) multigroup (*P*=0.0038, FDR=0.01, 55 variables. F-test) and (**b**) two-group comparison (*KMT2A-MLLT3*+Empty-GFP versus *KMT2A-MLLT3*+either *FLT3^{ITD}*, *FLT3^{N676K}*, or *NRAS^{G12D}*; *P*=0.001, FDR=0.01, 24 variables. F-test) of

mouse leukemias with or without an activating mutation using a variable list comprised of cytokines and growth factors (**Supplementary Data 29**).

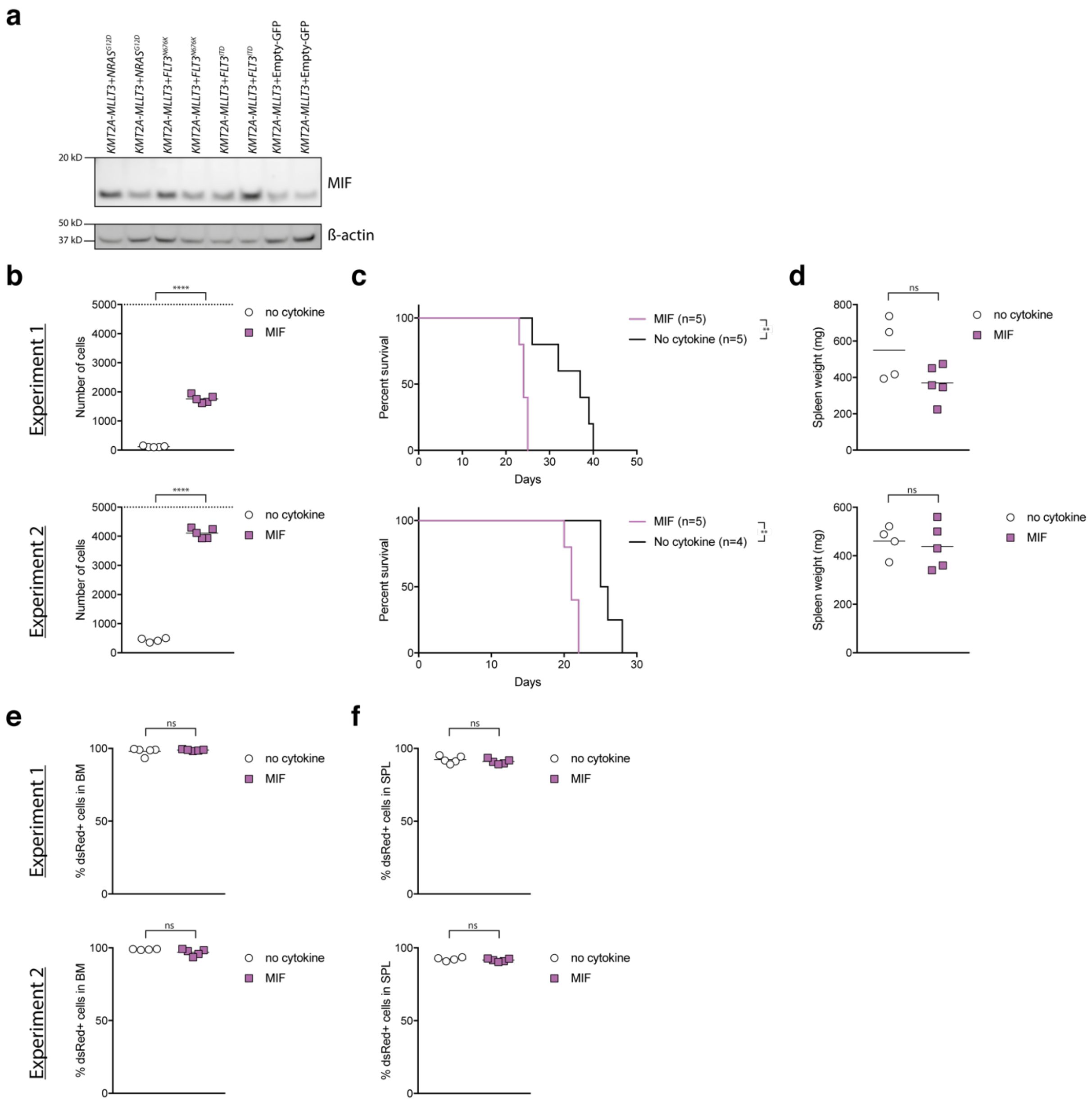

Supplementary Figure 11. MIF increase survival of *KMT2A-MLLT3* leukemia cell *ex vivo*. (**a**) Primary leukemia cells with or without activating mutations (dominant clones) were assessed for their relative protein levels of MIF. (**b-f**) Data for two experiments of recipient mice transplanted with serially propagated dsRed$^+$ *KMT2A-MLLT3* cells[6] cultured *ex vivo* for three days with or without 500 ng/ml MIF. (**b**) Number of leukemia cells[6] three days after *ex vivo* culture. (**c**) Kaplan-Meier survival curve showing the survival for transplanted recipient mice. (**d**) Spleen weight for recipient mice at the time of sacrifice. (**e**) Flow cytometric analysis of cells in BM and (**f**) spleen (SPL) from moribund mice transplanted with *ex vivo* cultured *KMT2A-MLLT3* leukemia cells.** $P\leq0.01$, **** $P\leq0.0001$, ns=not significant.

Supplementary Table 1: Genes included for targeted amplicon sequencing.

Andersson et. al. 2015 **and/or Lavallée et. al. 2015**	**Manually addded:**
Asxl1	*Bcr*
Atr	*Camk1d*
Braf	*Cdk13*
Cand2	*Kit*
Cbl	*Notch2*
Chd4	*Pdgfra*
Cp	*Pkn2*
Crebbp	*Runx1*
Flt3	*Wt1*
Idh2	
Jag1	
Jak2	
Kdm6a	
Kras	
L3mbtl3	
Nras	
Nsd1	
Pax5	
Pik3ca	
Pik3cd	
Pik3r1	
Plxna2	
Ptpn11	
Sfmbt2	
Spfi1	
Srsf2	
Stag2	
Supt3h	
Tet2	
Tet3	
Trp53	
Tubgcp6	

Supplementary Table 2: Sample distribution for targeted amplicon sequencing.

**Experimental group**	**Primary recipient**	**Matched Secondary recipient**
KMT2A-MLLT3 + Empty-GFP	28	8
KMT2A -MLLT3 + *FLT3-ITD* (dominant clone):	8	4
KMT2A -MLLT3 + *FLT3-N676K* (dominant clone):	11	7
KMT2A -MLLT3 + *NRAS-G12D* (dominant clone):	8	4
KMT2A -MLLT3 + *FLT3-N676K* (<50% BM clone):	7	6
**Total:**	62	29
Germline controls:	4	

Supplementary Table 3: Interphase fluorescence in situ hybridization for *Kras*.

	Number of signals (%)			
**Subject ID**	**1**	**2**	**3**	**4**
SJ016338	5 (4.6)	100 (92)	4 (3.7)	0 (0)
SJ046295	2 (0.9)	118 (55)	84 (39)	9 (4.2)

Supplementary Table 4: Sorting strategy for normal hematopoietic populations[1].

Sorted normal population	Gating strategy (pre-gated on live single lineage-depleted cells)
Hematopoietic stem cell (HSC)	$CD117^{+}$, Sca-1^{+}, $Flt3^{-}$, $CD34^{-}$, $CD150^{+}$
Multipotent myeloid progenitor (MPP)	$CD117^{+}$, Sca-1^{+}, $CD34^{+}$
Common myeloid progenitor (CMP)	$CD117^{+}$, Sca-1^{-}, $CD34^{+}$, $CD16/32^{low}$
Granulocyte monocyte progenitor (GMP)	$CD117^{+}$, Sca-1^{-}, $CD34^{+}$, $CD16/32^{+}$
Megakaryocyte erythroid progenitor (MEP)	$CD117^{+}$, Sca-1^{-}, $CD34^{-}$, $CD16/32^{-}$

Supplementary References

1. Dang, J. *et al.* AMKL chimeric transcription factors are potent inducers of leukemia. *Leukemia* **31,** 2228-2234 (2017).
2. Kim, J. *et al.* A Myc network accounts for similarities between embryonic stem and cancer cell transcription programs. *Cell* **143,** 313–324 (2010).
3. Yagi, T. *et al.* Identification of a gene expression signature associated with pediatric AML prognosis. *Blood* **102,** 1849–1856 (2003).
4. Zuber, J. *et al.* An integrated approach to dissecting oncogene addiction implicates a Myb-coordinated self-renewal program as essential for leukemia maintenance. *Genes Dev.* **25,** 1628–1640 (2011).
5. Pratilas, C. A. *et al.* (V600E)BRAF is associated with disabled feedback inhibition of RAF-MEK signaling and elevated transcriptional output of the pathway. *Proc. Natl. Acad. Sci. U.S.A.* **106,** 4519–4524 (2009).
6. Miller, P. G. *et al.* In Vivo RNAi screening identifies a leukemia-specific dependence on integrin beta 3 signaling. *Cancer Cell* **24,** 45–58 (2013).
