# Supplementary material for: De novo activating mutations drive clonal evolution and enhance clonal fitness in KMT2A-rearranged leukemia
Source: Nat Commun. 2018 May 2;9:1770. doi: 10.1038/s41467-018-04180-1 (PMC5932012; doi:10.1038/s41467-018-04180-1)
Supplement: Supplementary file 3 — Description of Additional Supplementary Files [file 41467_2018_4180_MOESM3_ESM.pdf]

Description of Additional Supplementary Files

File Name: Supplementary Data 1
Description: Coverage and uniformity for targeted amplicon sequencing.

File Name: Supplementary Data 2
Description: Identified de novo mutations from the targeted amplicon sequencing, their mutant allele frequency (MAF), and RNA sequencing reads confirming their expression.

File Name: Supplementary Data 3
Description: Leukemic samples subjected to RNA sequencing and number of total-, mapped-, and non-duplicate reads as well as percentage exonic coverage ≥1OX, ≥20X, and ≥30X. Also showing samples subjected to quantitative proteomics.

File Name: Supplementary Data 4
Description: Identified proteins and proteome data.

File Name: Supplementary Data 5
Description: GSEA results for *KMT2A-MLLT3* mouse leukemias with and without activating mutations compared to normal GMP using MSigDB c2all (RNA, FDR <0.05).

File Name: Supplementary Data 6
Description: Variable list composing the multigroup comparison heat map shown in Fig. 4a. Arranged in heat map order.

File Name: Supplementary Data 7
Description: GSEA results for *KMT2A-MLLT3* + Empty-GFP compared to *KMT2A-MLLT3* + Activating mutation using MSigDB c2all (RNA, FDR <0.05).

File Name: Supplementary Data 8
Description: GSEA results for *KMT2A-MLLT3* + Empty-GFP compared to *KMT2A-MLLT3* + $FLT3^{ITD}$ using MSigDB c2all (RNA, FDR <0.05).

File Name: Supplementary Data 9
Description: GSEA results for *KMT2A-MLLT3* + Empty-GFP compared to *KMT2A-MLLT3* + $FLT3^{N676K}$ using MSigDB c2all (RNA, FDR <0.05).

File Name: Supplementary Data 10
Description: GSEA results for *KMT2A-MLLT3* + Empty-GFP compared to *KMT2A-MLLT3* + $NRAS^{G12D}$ using MSigDB c2all (RNA, FDR <0.05).

File Name: Supplementary Data 11
Description: GSEA results for *KMT2A-MLLT3* + Empty-GFP compared to *KMT2A-MLLT3* + Activating mutation using MSigDB c2all (Protein, FDR <0.05).

File Name: Supplementary Data 12
Description: GSEA results for *KMT2A-MLLT3* + Empty-GFP compared to *KMT2A-MLLT3* + $FLT3^{ITD}$ using MSigDB c2all (Protein, FDR <0.05).

File Name: Supplementary Data 13
Description: GSEA results for *KMT2A-MLLT3* + Empty-GFP compared to *KMT2A-MLLT3* + $FLT3^{N676K}$ using

MSigDB c2all (Protein, FDR <0.05).

File Name: Supplementary Data 14
Description: GSEA results for *KMT2A-MLLT3* + Empty-GFP compared to *KMT2A-MLLT3* + *NRAS*^{G12D} using MSigDB c2all (Protein, FDR <0.05).

File Name: Supplementary Data 15
Description: GSEA custom gene sets used.

File Name: Supplementary Data 16
Description: GSEA results for *KMT2A-MLLT3* + Empty-GFP compared to *KMT2A-MLLT3* + Activating mutation using custom gene sets (RNA).

File Name: Supplementary Data 17
Description: GSEA results for *KMT2A-MLLT3* + Empty-GFP compared to *KMT2A-MLLT3* + *FLT3*^{ITD} using custom gene sets (RNA).

File Name: Supplementary Data 18
Description: GSEA results for *KMT2A-MLLT3* + Empty-GFP compared to *KMT2A-MLLT3* + *FLT3*^{N676K} using custom gene sets (RNA).

File Name: Supplementary Data 19
Description: GSEA results for *KMT2A-MLLT3*+Empty-GFP compared to *KMT2A-MLLT3* + *NRAS*^{G12D} using custom gene sets (RNA).

File Name: Supplementary Data 20
Description: GSEA results for *KMT2A-MLLT3* + Empty-GFP compared to *KMT2A-MLLT3* + Activating mutation using custom gene sets (Protein).

File Name: Supplementary Data 21
Description: GSEA results for *KMT2A-MLLT3* + Empty-GFP compared to *KMT2A-MLLT3*+ *FLT3*^{ITD} using custom gene sets (Protein).

File Name: Supplementary Data 22
Description: GSEA results for *KMT2A-MLLT3* + Empty-GFP compared to *KMT2A-MLLT3* + *FLT3*^{N676K} using custom gene sets (Protein).

File Name: Supplementary Data 23
Description: GSEA results for *KMT2A-MLLT3*+Empty-GFP compared to *KMT2A-MLLT3* + *NRAS*^{G12D} using custom gene sets (Protein).

File Name: Supplementary Data 24
Description: 2D-enrichment results for RNA- and Protein ratios between *KMT2A-MLLT3* + *FLT3*^{N676K} and *KMT2A-MLLT3* + Empty-GFP.

File Name: Supplementary Data 25
Description: 2D-enrichment results for RNA- and Protein ratios between *KMT2A-MLLT3* + *FLT3-N676K* and *KMT2A-MLLT3* + Empty-GFP.

File Name: Supplementary Data 26
Description: 2D-enrichment results for RNA- and Protein ratios between *KMT2A-MLLT3* + *NRAS*^{G12D} and *KMT2A-MLLT3* + Empty-GFP.

File Name: Supplementary Data 27
Description: GSEA results for *KMT2A-MLLT3* + Empty-GFP compared to *KMT2AMLLT3* + *Kras*^{G12D}/*Ptpn11*^{S506W} (MAF 0.41-0.59) using custom gene sets (RNA).

File Name: Supplementary Data 28
Description: GSEA results for infant ALL *KMT2A-AFF1* leukemia with- compared to without activating mutations (dominant and subclonal) using custom gene sets (RNA).

File Name: Supplementary Data 29
Description: Variable list of cytokines and growth factors present in the RNA sequencing.
